# Acknowledgements

**DOI:** 10.1002/ags3.12765

**Published:** 2024-01-19

The publication of invaluable papers in *Annals of Gastroenterological Surgery* depends on the prompt, careful review of submitted manuscripts. We would like to thank the following experts for reviewing manuscripts submitted between December 1, 2022 and November 30, 2023.

Abe, Yuta

Aizawa, Masaki

Ajiki, Tetsuo

Akamatsu, Nobuhisa

Akiyoshi, Takashi

Aoki, Takeshi

Aoki, Taku

Aoyama, Toru

Arigami, Takaaki

Arita, Junichi

Baba, Yoshifumi

Ban, Daisuke

Beppu, Naohito

Beppu, Toru

Bonavina, Luigi

Booka, Eisuke

Daiko, Hiroyuki

Ebata, Tomoki

Ebihara, Yuma

Eguchi, Hidetoshi

Etoh, Tsuyoshi

Fujii, Hodaka

Fujii, Tsutomu

Fujimura, Takashi

Fujita, Fumihiko

Fujiwara, Michitaka

Fujiwara, Toshiyoshi

Fujiwara, Yoshiyuki

Fukami, Yasuyuki

Fukatsu, Kazuhiko

Fukumoto, Takumi

Fukushima, Ryoji

Furuhata, Tomohisa

Gotohda, Naoto

Hanazaki, Kazuhiro

Harimoto, Norifumi

Hasegawa, Kiyoshi

Hasegawa, Suguru

Hashimoto, Daisuke

Hata, Taishi

Hatano, Etsuro

Hatao, Fumihiko

Hibi, Taizo

Hida, Koya

Hidaka, Masaaki

Higashi, Takahiro

Higuchi, Ryota

Hirahara, Noriyuki

Hisamatsu, Yuichi

Hiyoshi, Yukiharu

Honda, Goro

Hosoda, Kei

Iguchi, Tomohiro

Ikeda, Masataka

Ikeda, Satoshi

Ikegami, Toru

Ikoma, Hisashi

Imai, Katsunori

Imamura, Yu

Inaki, Noriyuki

Inokuchi, Mikito

Irino, Tomoyuki

Isaji, Shuji

Ishibashi, Nobuya

Ishido, Keinosuke

Ishiguro, Megumi

Ishihara, Soichiro

Ishizawa, Takeaki

Itabashi, Michio

Itano, Osamu

Ito, Takashi

Kagawa, Shunsuke

Kaibori, Masaki

Kaido, Toshimi

Kajiwara, Yoshiki

Kanda, Mitsuro

Kanemitsu, Yukihide

Kato, Motohiko

Kato, Yutaro

Kawachi, Shigeyuki

Kawai, Manabu

Kikuchi, Hirotoshi

Kimura, Yasutoshi

Kimura, Yutaka

Kinami, Shinichi

Kinoshita, Takahiro

Kitagawa, Akihiro

Kitago, Minoru

Kitayama, Joji

Kobayashi, Hirotoshi

Kobayashi, Minako

Kobayashi, Shogo

Koda, Keiji

Kojima, Kazuyuki

Kono, Koji

Kosuga, Toshiyuki

Koyanagi, Kazuo

Kubo, Shoji

Kubota, Takeshi

Kudo, Atsushi

Kumagai, Koshi

Kumagai, Youichi

Kumamaru, Hiraku

Kunisaki, Chikara

Kuriu, Yoshiaki

Kuroda, Shinji

Makino, Isamu

Marubashi, Shigeru

Matsuda, Keiji

Matsuda, Kenji

Matsuhashi, Nobuhisa

Matsumoto, Ippei

Matsuyama, Ryusei

Mayanagi, Shuhei

Miki, Yuichiro

Misawa, Takeyuki

Miura, Fumihiko

Miyamoto, Yuji

Mizuno, Shugo

Mizuno, Takashi

Monden, Kazuteru

Mori, Shinichirou

Morikawa, Takanori

Morine, Yuji

Morise, Zenichi

Motoi, Fuyuhiko

Motoyama, Satoru

Nagakawa, Yuichi

Naitoh, Takeshi

Nakada, Koji

Nakagawa, Kei

Nakamura, Masafumi

Namikawa, Tsutomu

Nanashima, Atsushi

Naziruddin, Bashoo

Niihara, Masahiro

Ninomiya, Itasu

Nishikawa, Kazuhiro

Nitta, Hiroyuki

Noda, Takehiro

Noma, Kazuhiro

Noshiro, Hirokazu

Nunobe, Souya

Obama, Kazutaka

Obara, Hideaki

Ohashi, Manabu

Ohtsuka, Masayuki

Ohtsuka, Takao

Okamoto, Koichi

Okano, Keiichi

Okugawa, Yoshinaga

Ono, Satoshi

Oshikiri, Taro

Oshima, Takashi

Ouchi, Akira

Rikitake, Ryoko

Saeki, Hiroshi

Saida, Yoshihisa

Saito, Takuro

Sakamoto, Yoshihiro

Sano, Tsuyoshi

Sasaki, Akira

Sato, Hiroshi

Satoi, Sohei

Seo, Satoru

Shibasaki, Susumu

Shibata, Chikashi

Shichinohe, Toshiaki

Shida, Dai

Shimada, Mitsuo

Shindo, Yoshitaro

Shinoda, Masahiro

Shinohara, Hisashi

Shinto, Eiji

Shiomi, Akio

Shiozaki, Atsushi

Shoda, Katsutoshi

Soejima, Yuji

Suda, Koichi

Sugiura, Teiichi

Suwa, Yusuke

Takada, Yasutsugu

Takahara, Takeshi

Takahashi, Hidekazu

Takahashi, Hidenori

Takahashi, Takao

Takahashi, Tsuyoshi

Takatsuki, Mitsuhisa

Takeishi, Kazuki

Takeno, Shinsuke

Taketomi, Akinobu

Tanaka, Hiroaki

Tanaka, Keitaro

Tanaka, Koji

Tani, Masaji

Taura, Kojiro

Terashima, Masanori

Toh, Yasushi

Tokumitsu, Yukio

Tokunaga, Masanori

Tomimaru, Yoshito

Toyonaga, Takashi

Tsukamoto, Shunsuke

Uemura, Kenichiro

Uemura, Mamoru

Uetake, Hiroyuki

Umeda, Yuzo

Wakai, Toshifumi

Wakasugi, Masaki

Wakiya, Taiichi

Watanabe, Hiroaki

Watanabe, Manabu

Watanabe, Masayuki

Yachida, Shinichi

Yamada, Kazuhiko

Yamada, Suguru

Yamada, Takanobu

Yamaguchi, Tomohiro

Yamamoto, Kazuyoshi

Yamamoto, Seiichiro

Yamamoto, Yusuke

Yamashita, Yo‐Ichi

Yanagimoto, Yoshitomo

Yasuda, Takushi

Yasui, Masayoshi

Yoh, Tomoaki

Yoshida, Naoya

Yoshikawa, Kozo

Yoshikawa, Takaki

Yoshimatsu, Kazuhiko

Yoshitomi, Hideyuki

